# Grey Language Hesitant Fuzzy Group Decision Making Method Based on Kernel and Grey Scale

**DOI:** 10.3390/ijerph15030436

**Published:** 2018-03-02

**Authors:** Qingsheng Li, Yuzhu Diao, Zaiwu Gong, Aqin Hu

**Affiliations:** School of Business, Linyi University, Linyi 276000, China; diaoyuzhu@126.com (Y.D.); zwgong26@163.com (Z.G.); huaqin@lyu.edu.cn (A.H.)

**Keywords:** kernel and grey scale, grey language, hesitant fuzzy, grey correlation

## Abstract

Based on grey language multi-attribute group decision making, a kernel and grey scale scoring function is put forward according to the definition of grey language and the meaning of the kernel and grey scale. The function introduces grey scale into the decision-making method to avoid information distortion. This method is applied to the grey language hesitant fuzzy group decision making, and the grey correlation degree is used to sort the schemes. The effectiveness and practicability of the decision-making method are further verified by the industry chain sustainable development ability evaluation example of a circular economy. Moreover, its simplicity and feasibility are verified by comparing it with the traditional grey language decision-making method and the grey language hesitant fuzzy weighted arithmetic averaging (GLHWAA) operator integration method after determining the index weight based on the grey correlation.

## 1. Introduction

The deepening interdependence and mutual influence of the world economy and the application of the new generation of Internet information technology have significantly influenced the decisions of the state and enterprises. The complexity, uncertainty and collectivization of decision-making information in politics, the economy and military have become the new normal of decision making. 

A complex decision-making environment and the limited cognitive ability of decision makers require overcoming the constraints of traditional sets. In 1965, the American cybernetics expert Zadeh [1] first proposed the concept of the fuzzy set, which expresses the idea of uncertainty through membership degree and has been widely used in various fields, such as management decisions, medical diagnosis and engineering control. The Bulgarian scholar Atanassov [2] argued that the membership degree of traditional fuzzy sets can only be the numbers between 0 and 1, which cannot dig deeper into the fuzzy degree in actual decision making. Based on the above consideration, Atanassov studied three dimensions of membership degree, non-membership degree and hesitation degree, and gave the relational expression, which was further developed into an intuitive fuzzy set. Since Zadeh proposed fuzzy sets, many scholars have studied their extension forms, such as the intuitionistic fuzzy set (IFS) [3], the type 2 fuzzy set [4,5], the fuzzy multiple set, and [6,7] the hesitant fuzzy set. This includes group decision making based on information integration and the measurement of hesitant fuzzy sets. Based on Archimedes T-module and S-module, Xia Meimei gave the rules of hesitant fuzzy operation and the information integration operator of hesitant fuzzy decision; defined the distance, similarity, correlation, entropy and cross-entropy of hesitant fuzzy sets; and discussed their properties. Moreover, their correlation was studied in order to apply this to group decision-making problems [8]. These studies have laid a foundation for the study of hesitant fuzzy group decision making based on the kernel and gray level.

Increasingly complex decision information requires experts to make group decisions in more cases. Experts need to face the complex environment with both complete and incomplete information, both definite information and uncertain information. The complex group decision-making environment has also attracted the attention of domestic scholars. In 1982, Deng Julong, a Chinese scholar, published the first paper about the grey system entitled “The Control Problems of Grey Systems in Systems and Control Letters” [9]. In the same year, Deng Julong published the first Chinese grey system paper “Grey Control System” in the Journal of the Huazhong Institute of Technology [10]. These two innovative classical papers have aroused wide attention and successfully initiated an emerging trans-disciplinary science—grey system theory [11]. 

The grey multi-attribute decision-making method was studied by [12,13,14,15]. Youchen et al. studied the inverse order problem of the grey decision model [16]. Zhang Na et al. established the multi-stage grey situation group decision model based on the Orness measure constraint [17]. Liu Sifeng et al. explored the two-stage grey comprehensive measurement decision model and the improvement of the triangular whitening weight function [18]. Luo Dang and Liu Sifeng studied the grey relational decision method of the grey relational decision method and an incomplete information system [19,20]. Dang Yaoguo et al. studied the grey relational decision model based on a dynamic multi-index [21]. Jiang Shiquan et al. put forward a grey relational decision model based on area [22]. Xie Ming et al. studied the grey relational analysis method of multi-attribute group decision making based on the analytic hierarchy process (AHP) and its application [23]. Chen Xiaoxin and Liu Sifeng put forward a grey relational decision method with partial weight information and a preference to scheme [24]. The rapid development of the grey relational group decision-making method provides the research foundation for the fuzzy grey relational decision making in this paper. 

The differences of knowledge level, judgment experience and preference of decision experts will inevitably lead to the deviation of information interpretation, which further causes uncertain decision-making problems accompanied by fuzzy and grey characteristics, namely, the grey fuzzy multi-attribute group decision-making problem. The intuitionistic fuzzy decision making method was studied by [25,26,27,28,29] on the basis of grey correlation combined with evidence reasoning and the expert system uncertainty factor. Hu Lifang et al. organically combined grey with fuzzy, and put forward the grey fuzzy multi-attribute decision making method under the frame of the closed world [30]. Li Peng et al. put forward the combination method of grey relation and Dempster-Shafer evidence theory, which was applied to multiple-criteria decision making (MCDM) based on the intuitionistic fuzzy set (IFS) [31]. 

Some scholars from Nanjing University of Aeronautics and Astronautics combined grey system with fuzzy theory, and put forward relevant decision methods. For example, Li Peng, Liu Sifeng and Zhu Jianjun studied the combination of GR, DS and MYCIN, which was applied to IFS decision-making [32,33]. Liu Yong and Liu Sifeng studied the dynamic multi-attribute grey relational decision making method based on interval intuitionistic fuzzy [26]. In [34], the grey correlation method and non-linear programming model were used to obtain the optimal weight and study the relevant decision methods. The study of grey system and fuzzy combination promotes the fusion and promotion of the uncertainty decision method.

Research in the field of grey language multi-attribute decision-making is relatively scarce. Wu Jianwen studied the grey language multi-criteria decision method and its application [35]. Wang Jiang et al. put forward a multi-criteria decision method based on interval grey uncertain language [36]. Liu Peide et al. proposed a multi-attribute group decision-making method based on a geometric weighted integration operator of interval grey language variables [37]. Wang Hehua studied the information aggregation model of language evaluation based on grey decision and its application [38]. Li Qingsheng et al. [39,40,41] put forward the grey hesitant fuzzy set and extended it to the grey language form, namely, the grey language hesitant fuzzy set, which can conveniently use the special decision method of a grey system and effectively solve the problem of hesitant fuzzy decision making. How to take the advantages of a grey system to make grey language hesitant fuzzy decision expand the application scope of a grey system is significant for enriching and perfecting grey system theory and the organic combination of uncertain decision making. This paper proposes a kernel and grey scale-scoring function of the grey language hesitant fuzzy set which uses the grey correlation degree to make decisions.

## 2. Preliminaries 

### 2.1. Grey Language Set

The concepts of language value and grey scale are generally combined in some practical decision-making processes, so that the decision information is more in line with the decision habit, which is more like the description of kernel and grey scale based on a grey system. Wu Jianwen and Wang Jianjiang put forward the grey language set by studying the multi-criteria decision and grey system of language, and discussed the problem of multi-attribute decision-making based on grey language. For example, a college counselor gives the evaluation results of “excellent”, “good”, “fair”, “qualified” and “failing”. If a college student is rated as “good”, the membership degree of the comprehensive evaluation value of “good” is 1. However, the language evaluation value of membership degree of 1 is often impractical, as it requires counselors to grasp comprehensive information about the student, including learning, interpersonal relations, comprehensive ability in the family, society, classroom and dormitory, etc. Obviously, this is impossible in reality. The decision maker’s “limited rationality” principle proposed by Simon is closer to reality. If the counselor can give the membership degree and incomplete degree of information (grey scale), it will be very helpful for the scientific evaluation of college students. Therefore, evaluation values in the following form can be obtained: $A=\left\{x,H_{i},\mu_{i},v_{i}|x\in X\right\}=\left\{x,good,0.8,0.4\right\}$, which represents the membership degree to which the college student is evaluated as “good” at 0.8, and the information uncertainty at 0.4.

#### 2.1.1. Definition of Grey Language Set

**Definition** **1.** *Set the discourse domain* X, *and language phase set* $H=\left\{H_{0},H_{1},H_{2},\cdots H_{T-1},H_{T}\right\}$. *For* ∀x∈X, *there is a corresponding language value* $H_{i}(H_{i}\in H)$. *If the membership degree of x to* $H_{i}$ *is the grey number in [0,1], its point grey scale is* v(x), $A=\left\{x,H_{i},\mu_{i},v_{i}|x\in X\right\}$ *is a grey language set (*GLS) *on* X. $\mu_{H_{i}}(x)$ *is the membership degree, which can be understood as the close degree of* x *to* $H_{i}$ *[38]. Different from intuitionistic fuzzy sets*,v(x) *represents the extent to which the decision maker's information is incomplete, namely, grey scale, instead of non-membership degree. Although there is no necessary correlation between grey scale* v(x) *and membership degree* $\mu_{H_{i}}(x)$, *the smaller the value of* $\left|\mu_{i}+v_{i}-1\right|$, *the better the reliability*.

In general, the grey language set A is abbreviated as A={H(x),μ(x),v(x)|x∈X}. If a={H(x),μ(x),v(x)} is GLS, any specific grey language element a in A can be abbreviated as GLE. If the values of membership degree and grey scale in a is μ(x)=1,v(x)=0, a does not have incomplete information degree, and retreats into the general language number.

#### 2.1.2. Algorithm of Grey Language Number 

(1) Set $a_{i}=\left\{H_{i},\mu_{i},v_{i}\right\}$ and $a_{j}=\left\{H_{j},\mu_{j},v_{j}\right\}$ as arbitrary GLE, according to the following rules [35].

$a_{i}⊕a_{j}=(H_{i}⊕H_{j},\frac{i\cdot \mu_{i}+j\cdot \mu_{j}}{i+j},\max(v_{i},v_{j}))$, when $H_{i}$ and $H_{j}$ are not equal to $H_{0}$ simultaneously.

$a_{i}⊕a_{j}=(H_{i}⊕H_{j},\max(\mu_{i},\mu_{j}),\max(v_{i},v_{j}))$, when $H_{i}$ and $H_{j}$ are equal to $H_{0}$. In the expression and calculation process of grey language “⊕” in this paper, if there is no special explanation, it is operated as $H_{i}$ and $H_{j}$ are not equal to $H_{0}$ simultaneously.

(2) $ra_{i}=\left\{rH_{i},\mu_{i},v_{i}\right\}$ [35].

(3) $a_{i}⊗a_{j}=\left(H_{i}⊗H_{j},\mu_{i}\cdot \mu_{j},v_{i}+v_{j}-v_{i}\cdot v_{j}\right)$ [35].

(4) $a_{i}^{\lambda }=\left(H_{i}^{\lambda },\mu_{i}^{\lambda },1-{\left(1-v_{i}\right)}^{\lambda }\right)$ [35].

#### 2.1.3. Properties of Grey Language Number Algorithm

Assume that $a_{i}=\left\{H_{i},\mu_{i},v_{i}\right\}$, $a_{j}=\left\{H_{j},\mu_{j},v_{j}\right\}$ and $a_{k}=\left\{H_{k},\mu_{k},v_{k}\right\}$ are any three grey language numbers, and the arithmetic operation has the following properties:

(1) $a_{i}⊕a_{j}=a_{j}⊕a_{i}$.

(2) $\left(a_{i}⊕a_{j})a_{k}=a_{i}⊕(a_{j}⊕a_{k}\right)$.

(3) $a_{i}⊗a_{j}=a_{j}⊗a_{i}$.

(4) $\left(a_{i}⊗a_{j})a_{k}=a_{i}⊗(a_{j}⊗a_{k}\right)$.

(5) $\lambda a_{i}⊕\lambda a_{j}=\lambda (a_{i}⊕a_{j}),\lambda >0$.

(6) $\lambda_{1}a_{i}⊕\lambda_{2}a_{i}=(\lambda_{1}+\lambda_{2})a_{i},\lambda_{1}>0,\lambda_{2}>0$.

(7) ${\left(a_{i}⊗a_{j}\right)}^{\lambda }=a_{i}{}^{\lambda }⊗a_{j}{}^{\lambda },\lambda \geq 0$.

(8) $a_{i}{}^{\lambda_{1}}⊗a_{i}{}^{\lambda_{2}}=a_{i}{}^{\left(\lambda_{1}+\lambda_{2}\right)},\lambda_{1}\geq 0,\lambda_{2}\geq 0$.

#### 2.1.4. Size of Grey Language Numbers

**Definition** **2.** *Assume* $a_{i}=\left\{H_{i},\mu_{i},v_{i}\right\}$ *and* $a_{j}=\left\{H_{j},\mu_{j},v_{j}\right\}$ *are any two grey language numbers [38]. If* $(1-v_{i})\cdot \mu_{i}\cdot IH_{i}<(1-v_{j})\cdot \mu_{j}\cdot IH_{j}$, $a_{i}$ *is smaller than* $a_{j}$, *denoted as* $a_{i}<a_{j}$;

*If* $(1-v_{i})\cdot \mu_{i}\cdot IH_{i}>(1-v_{j})\cdot \mu_{j}\cdot IH_{j}$, $a_{i}$ *is greater than* $a_{j}$, *denoted as* $a_{i}>a_{j}$;

*If* $(1-v_{i})\cdot \mu_{i}\cdot IH_{i}=(1-v_{j})\cdot \mu_{j}\cdot IH_{j}$, *and* $H_{i}=H_{j},\mu_{i}=\mu_{j},v_{i}=v_{j}$, $a_{i}$ *is strongly equal to* $a_{j}$, *denoted as* $a_{i}=a_{j}$;

*If* $(1-v_{i})\cdot \mu_{i}\cdot IH_{i}=(1-v_{j})\cdot \mu_{j}\cdot IH_{j}$, *and at least one of* $H_{i}=H_{j},\mu_{i}=\mu_{j},v_{i}=v_{j}$, *is tenable*, $a_{i}$ *is weakly equal to* $a_{j}$, *denoted as* $a_{i}\approx a_{j}$. *Where*, I *is subscript operator*.

### 2.2. Hesitant Fuzzy Set

When the attribute of a given scheme belongs to the membership degree of a certain set, it is not necessarily a random interval nor does it obey a certain distribution. The performance will be more prominent in group decision making. The membership degree given by different groups may be uncoordinated, and sometimes it is not necessary to reach consistent possible values. To this end, Torra [42,43] proposed the hesitant fuzzy set which can better meet the needs of group decision making.

#### 2.2.1. Definitions Related to Hesitant Fuzzy Sets

**Definition** **3.** *Assume* X *is a given set, and hesitant fuzzy set is the projection from* X → *[0,1], abbreviated as* A={〈x,h(x)〉|x∈X}. *Where*, h(x) *is the set of several hesitant fuzzy numbers (HFN) in [0,1]*. HFN *is the membership degree value of* x∈X *belonging to set A. Obviously, there is 0≤HFN≤1 [42,43].*

The hesitant fuzzy element contains the possible hesitant fuzzy number in the set. The length of data in each hesitant fuzzy element is likely to vary. The following method is used to determine the size of the hesitant fuzzy element.

**Definition** **4.** *Any hesitation fuzzy element* h, $s(h)=1/l\sum_{1}^{l}{}_{\gamma \in h}\gamma$ *is score function, where* l *is the number of elements in* h. *There are two hesitant fuzzy elements* $h_{1},h_{2}$. *If* $s(h_{1})>s(h_{2})$, $h_{1}>h_{2}$. *If* $s(h_{1})=s(h_{2})$, $h_{1}\approx h_{2}$.

Torra believed that membership degree at both ends of hesitant fuzzy can be understood as an intuitionistic fuzzy number.

**Definition** **5.** *The envelope of any hesitant fuzzy number* h *can be converted to intuitionistic fuzzy number through the following formula: $A_{env}(h)=\left(h^{-},(1-h^{+})\right)$, where $h^{-}=\min\gamma ,h^{+}=\max\gamma \begin{array}{cc} & \gamma \in h \end{array}$ [42,43].*

Based on the connotation of hesitant fuzzy envelope relation [8] studied the envelop relation set operation.

(1) $A_{env}(h^{c})={\left(A_{env}(h)\right)}^{c}$.

(2) $A_{env}(h_{1}\cup h_{2})=A_{env}(h_{1})\cup A_{env}(h_{2})$.

(3) $A_{env}(h_{1}\cap h_{2})=A_{env}(h_{1})\cap A_{env}(h_{2})$.

(4) $A_{env}(h^{\lambda })={\left(A_{env}(h)\right)}^{\lambda }$.

(5) $A_{env}(\lambda h)=\lambda A_{env}(h)$.

(6) $A_{env}(h_{1}\cdot h_{2})=A_{env}(h_{1})\cdot A_{env}(h_{2})$.

(7) $A_{env}(h_{1}+h_{2})=A_{env}(h_{1})+A_{env}(h_{2})$.

#### 2.2.2. Algorithms of Hesitant Fuzzy Set

Assume any three hesitant fuzzy numbers $h,h_{1},h_{2}$, and Torra [42,43] studied their algorithms:

(1) $h^{c}=\cup_{\gamma \in h}\left\{1-\gamma \right\}$.

(2) $h_{1}\cup h_{2}=\cup_{\gamma_{1}\in h_{1},\gamma_{2}\in h_{2}}\max\left\{\gamma_{1},\gamma_{2}\right\}$.

(3) $h_{1}\cap h_{2}=\cup_{\gamma_{1}\in h_{1},\gamma_{2}\in h_{2}}\min\left\{\gamma_{1},\gamma_{2}\right\}$.

On the basis of studying the operation of Archimedes T-module and S-module [8] studied the algorithm of hesitant fuzzy operation. There are arbitrary hesitant fuzzy elements $h,h_{1},h_{2}$.

(1) $h^{\lambda }=\cup_{\gamma \in h}\left\{k^{-1}\left(\lambda k(\gamma )\right)\right\}$.
(2) $\lambda h=\cup_{\gamma \in h}\left\{l^{-1}\left(\lambda l(\gamma )\right)\right\}$.
(3) $h_{1}\cdot h_{2}=\cup_{\gamma_{1}\in h_{1},\gamma_{2}\in h_{2}}\left\{k^{-1}\left(k(\gamma_{1})+k(\gamma_{2})\right)\right\}$.
(4) $h_{1}+h_{2}=\cup_{\gamma_{1}\in h_{1},\gamma_{2}\in h_{2}}\left\{l^{-1}\left(l(\gamma_{1})+l(\gamma_{2})\right)\right\}$.

where l(t)=k(1−t), and k:[0,1]→[0,+∞) is a strictly decreasing function.

The hesitant fuzzy operation rule can be defined as below:

**Definition** **6.** *There are three hesitant fuzzy numbers* $h,h_{1},h_{2}$.

(1) ${\left(h^{c}\right)}^{\lambda }={\left(\lambda h\right)}^{c}$.
(2) $\lambda (h^{c})={\left(h^{\lambda }\right)}^{c}$.
(3) $h_{1}^{c}\cdot h_{2}^{c}={\left(h_{1}^{}+h_{2}^{}\right)}^{c}$.
(4) $h_{1}^{c}+h_{2}^{c}={\left(h_{1}^{}\cdot h_{2}^{}\right)}^{c}$.

where, $h^{c}=\cup_{\gamma \in h}\left\{1-\gamma \right\}$ [8].

**Definition** **7.** *Assume* $h_{i}(i=1,2,\cdots ,n)$ *as a set of hesitant fuzzy numbers*, $\omega ={\left(\omega_{1},\omega_{2},\cdots ,\omega_{n}\right)}^{T}$ *satisfie*, $\sum_{1}^{n}\omega_{i}=1,\begin{array}{cc} \omega_{i}\geq 0, & \left(i=1,2,\cdots ,n\right) \end{array}$, $ATS-HFWA(h_{1},h_{2},,h_{n})=\sum_{i=1}^{n}\omega_{i}\cdot h_{i}^{}$.

ATS−HFWA is a hesitant fuzzy weighted arithmetic averaging operator based on Archimedes T-Module and S-Module.

$$ATS-HFWG(h_{1},h_{2},\cdots ,h_{n})=\prod_{i=1}^{n}{h_{i}}^{\omega_{i}}.$$

ATS−HFWG is a hesitant fuzzy weighted geometric averaging operator based on Archimedes T-Module and S-Module [8].

### 2.3. Kernel and Grey Scale

#### 2.3.1. Definitions of Kernel and Grey Scale

The study of grey number operation in grey system theory has attracted attention for a long period of time, but no satisfactory result has been achieved.

**Definition** **8.** *Let the grey number* $⊗\in [\underline{a},\bar{a}],\underline{a}<\bar{a}$ *in the absence of grey number value distribution information [44]:*

*If* ⊗ *is a continuous grey number*, $\hat{⊗}=\frac{1}{2}(\underline{a}+\bar{a})$ *is the kernel of grey number* ⊗.

*If* ⊗ *is discrete grey number*, $a_{i}\in [\underline{a},\bar{a}],(i=1,2,\cdots ,n)$ *is the possible value of grey number. Its average value is the kernel of grey number, namely*, $\hat{⊗}=\frac{1}{n}\sum_{i=1}^{n}a_{i}$ *is the kernel of grey number* ⊗.

**Definition** **9.** *If* ⊗ *is a random grey number, and the mathematical expectation* E(⊗) *can be calculated*, E(⊗) *is the kernel of random* ⊗ *and can expressed as $\hat{⊗}=E(⊗)$ [44].*

**Definition** **10.** *Set the background or all of the grey number* ⊗ *is* Ω,μ(⊗) *as the measure of interval grey number* ⊗ *taking the number field*. $g^{o}(⊗)=\mu (⊗)/\mu (\Omega )$ *is the grey level of grey number* ⊗, *which can be understood as the ratio of all possible membership degree to background, abbreviated as $g^{o}$ [44].*

The properties of ⊗∈Ω and the measure show that the grey scale satisfies the following properties: grey scale satisfies the criterion, namely, $0\leq g^{o}(⊗)\leq 1$.

When $g^{o}=0$, the uncertainty is 0, which is the white number for complete certainty;

When $g^{o}=1$, the uncertainty is 1, which is the black number for complete uncertainty;

When $0<g^{o}(⊗)<1$, it is an uncertain grey number.

The grey scale reflects the uncertainty degree of the things described by the grey number. The white number is a completely certain number, and the black number is completely unknown. When $g^{o}$ is closer to 0, the uncertainty of the grey number is smaller. When $g^{o}$ is closer 1, the uncertainty of the value range is greater.

**Definition** **11.** $\hat{⊗}$ *is the kernel of grey number* ⊗, *and* $g^{o}$ *is the grey scale*. ${\hat{⊗}}_{g^{o}}$ *is the simplified form of grey numbers*. ${\hat{⊗}}_{g^{o}}$ *includes all information of value, and has one-to-one correspondence [44].*

On the one hand, ${\hat{⊗}}_{g^{o}}$ can be calculated according to the definition of the kernel and grey scale. On the other hand, the range of ⊗ can also be calculated according to ${\hat{⊗}}_{g^{o}}$ [45] proposed the algorithm based on $\hat{⊗}$ and $g^{o}$ and the grey scale non-decreasing axiom.

#### 2.3.2. Algorithms of Kernel and Grey Scale

**Axiom** **1.** Grey scale non-decreasing axiom: grey scale is equal to the maximum grey level of all grey numbers involved in the operation, and it will not decrease [44].

According to Axiom 1, the simplified form ${\tilde{⊗}}_{k}(g_{k}^{o})$ based on the grey number can obtain the grey data algebraic algorithm [45].

(1) ${\tilde{⊗}}_{k}(g_{k}^{o})+{\tilde{⊗}}_{m}(g_{m}^{o})=({\tilde{⊗}}_{k}+{\tilde{⊗}}_{m})(g_{k}^{o}(\vee g_{m}^{o})$.

(2) ${\tilde{⊗}}_{k}(g_{k}^{o})-{\tilde{⊗}}_{m}(g_{m}^{o})=({\tilde{⊗}}_{k}-{\tilde{⊗}}_{m})(g_{k}^{o}\vee g_{m}^{o})$.

(3) ${\tilde{⊗}}_{k}(g_{k}^{o})\times {\tilde{⊗}}_{m}(g_{m}^{o})=({\tilde{⊗}}_{k}\times {\tilde{⊗}}_{m})(g_{k}^{o}(\vee g_{m}^{o})$.

(4) ${\tilde{⊗}}_{k}(g_{k}^{o})÷{\tilde{⊗}}_{m}(g_{m}^{o})=({\tilde{⊗}}_{k}÷{\tilde{⊗}}_{m})(g_{k}^{o}(\vee g_{m}^{o})$ $\left({\tilde{⊗}}_{m}\neq 0\right)$.

(5) $m\times {\tilde{⊗}}_{k}(g_{k}^{o})=(m\times {\tilde{⊗}}_{k})(g_{k}^{o})$.

(6) ${\left({\tilde{⊗}}_{k}(g_{k}^{o})\right)}^{r}={\left({\tilde{⊗}}_{k}\right)}^{r}(g_{k}^{o})$.

Grey heterogeneous data may be included by the interval grey number, discrete grey number, real number or other grey information. All the different data structures and grey information characteristics belong to the same “grey” category, have a common attribute “kernel” and “grey scale” (real is a special grey number whose grey scale is “0”). So the algebraic algorithm between grey heterogeneous data can be studied through the “kernel” and “grey scale”.

**Definition** **12.** *Assume* F(⊗) *is a grey heterogeneous data set. For arbitrary* ${⊗}_{i}$ *and* ${⊗}_{j}$, ${⊗}_{i}+{⊗}_{j}$, ${⊗}_{i}-{⊗}_{j}$, ${⊗}_{i}\times {⊗}_{j}$ *and* ${⊗}_{i}÷{⊗}_{j}({⊗}_{j}\neq 0)$ *belong to* F(⊗)*, and is grey heterogeneous data field [46].*

Assume F(⊗) as the grey heterogeneous data set. ${⊗}_{i}$, ${⊗}_{j}$ and ${⊗}_{k}$ ∈F(⊗). The kernel and grey scale are ${\tilde{⊗}}_{i}(g_{i}^{o})$,${\tilde{⊗}}_{j}(g_{j}^{o})$ and ${\tilde{⊗}}_{k}(g_{k}^{o})$. Grey heterogeneous data algebraic algorithms have the following properties [45]: 

(1) ${\tilde{⊗}}_{i}(g_{i}^{o})+{\tilde{⊗}}_{j}(g_{j}^{o})={\tilde{⊗}}_{j}(g_{j}^{o})+{\tilde{⊗}}_{i}(g_{i}^{o})$.

(2) $\left({\tilde{⊗}}_{i}(g_{i}^{o})+{\tilde{⊗}}_{j}(g_{j}^{o}))+{\tilde{⊗}}_{k}(g_{k}^{o})={\tilde{⊗}}_{i}(g_{i}^{o})+({\tilde{⊗}}_{j}(g_{j}^{o})+{\tilde{⊗}}_{k}(g_{k}^{o})\right)$.

(3) ∃∀0∈F(⊗), Make ${\tilde{⊗}}_{i}(g_{i}^{o})+0={\tilde{⊗}}_{i}(g_{i}^{o})$.

(4) For $\forall {⊗}_{s}\in F(⊗)$, $-{⊗}_{s}\in F(⊗)$ and make $-{⊗}_{s}+{⊗}_{s}=0$.

(5) ${\tilde{⊗}}_{i}(g_{i}^{o})\times ({\tilde{⊗}}_{j}(g_{j}^{o})\times {\tilde{⊗}}_{k}(g_{k}^{o}))=({\tilde{⊗}}_{i}(g_{i}^{o})\times {\tilde{⊗}}_{j}(g_{j}^{o}))\times {\tilde{⊗}}_{k}(g_{k}^{o})$.

(6) There is unit element 1∈F(⊗) which makes $1\times {\tilde{⊗}}_{i}(g_{i}^{o})={\tilde{⊗}}_{i}(g_{i}^{o})\times 1={\tilde{⊗}}_{i}(g_{i}^{o})$.

(7) $\left({\tilde{⊗}}_{i}(g_{i}^{o})+{\tilde{⊗}}_{j}(g_{j}^{o}))\times {\tilde{⊗}}_{k}={\tilde{⊗}}_{i}(g_{i}^{o})\times {\tilde{⊗}}_{k}(g_{k}^{o})+{\tilde{⊗}}_{j}(g_{j}^{o})\times {\tilde{⊗}}_{k}(g_{k}^{o}\right)$.

(8) ${\tilde{⊗}}_{i}(g_{i}^{o})\times ({\tilde{⊗}}_{j}(g_{j}^{o})+{\tilde{⊗}}_{k}(g_{k}^{o}))={\tilde{⊗}}_{i}(g_{i}^{o})\times {\tilde{⊗}}_{j}(g_{j}^{o})+{\tilde{⊗}}_{i}(g_{i}^{o})\times {\tilde{⊗}}_{k}(g_{k}^{o})$.

## 3. Grey Language Hesitant Fuzzy Information Aggregation and Kernel and Grey Scale Scoring Function

### 3.1. Information Aggregation of Grey Language Hesitant Fuzzy Decision

Since the evaluation criteria are given in the form of grey language, the number l of decision experts is the length of the data, which is denoted as $r_{ij}=\left\{\left(H_{\sigma 1},\mu_{1},v_{1}),(H_{\sigma 2},\mu_{2},v_{2}),\cdots (H_{\sigma l},\mu_{l},v_{l}\right)\right\}$, where σk is the IH value of the *k*th item. The data are aggregated by the algorithm of the grey language fuzzy number to obtain $z_{ij}$.

(1) $$z_{ij}=(H_{\frac{1}{l}(\sum_{k=1}^{l}\sigma k)},\frac{\sigma 1\cdot \mu_{1}+\sigma 2\cdot \mu_{2}+\cdots \sigma l\cdot \mu_{l}}{\sum_{k=1}^{l}\sigma k},\max(v_{1},v_{2}\cdots v_{l}))$$

**Definition** **13.** *Assume* $A=\left\{a_{k}|k=1,2,3\cdots l\right\}$ *as the grey language set*, $a_{k}=\left\{H_{k},\mu_{k},v_{k}\right\}$ *and* $g(a_{1},a_{2},a_{3},\cdots a_{l})=\sum_{k=1}^{l}w_{k}a_{k}$.

Where, $w_{k}$ is the weight vector corresponding to the grey language array $a_{k}$.$w_{k}\in [0,1]$ and $\sum_{k=1}^{l}w_{k}=1$, g is grey language weighted average operator, abbreviated as the GLHFWAA operator.

(2) $$GLHFWAA(a_{1},a_{2},\cdots a_{l})=\sum_{k=1}^{l}w_{k}a_{k}=\sum_{k=1}^{l}(w_{k}H_{k},\mu_{k},v_{k})$$

If $w=\left(\frac{1}{l},\frac{1}{l},\cdots ,\frac{1}{l}\right)$, GLHFWAA degenerates to grey language averaging operator GLAA,

$$GLHFAA(a_{1},a_{2},\cdots a_{l})=\sum_{k=1}^{l}w_{k}a_{k}=\sum_{k=1}^{l}(\frac{1}{l}H_{k},\mu_{k},v_{k}).$$

### 3.2. Grey Language Hesitant Fuzzy Score Function

**Definition** **14.** *Assume* $a_{i}=\left\{H_{i},\mu_{i},v_{i}\right\}$ *as any grey language number, and* $S(a_{k})$ *is the score function of* $a_{k}$.

(3) $$S(a_{k})=(1-v_{k})\cdot \mu_{k}\cdot IH_{k}$$

Assume glh∈GLH, $GLH=\left\{\left(H_{1},\mu_{1},v_{1}),(H_{2},\mu_{2},v_{2}),\cdots (H_{l},\mu_{l},v_{l}\right)\right\}$ are grey language decision data given by l experts.

(4) $$S(GLH)=\frac{1}{l}\sum_{k=1}^{l}S(a_{k})\begin{array}{cc} & k=1,2\cdots l \end{array}$$

S(GLH) is the score function of GLH.

Example. Let GLH={(3,0.6,0.2),(4,0.6,0.3),⋯(4,0.7,0.2)}, $S(GLH)=\frac{1}{l}\sum_{k=1}^{l}S(a_{k})=\frac{1}{3}\times \left[(1-0.2)\times 0.6\times 3+(1-0.3)\times 0.6\times 4+(1-0.2)\times 0.7\times 4\right]=1.7867$.

The above definition transforms uncertain grey language information into a score function and becomes a definite value, which is theoretically inadequate. In this paper, it is considered that the score function should be a grey number [45] believed that the kernel $\hat{⊗}$ of the grey number is an important representative of the value of the grey scale ⊗. Kernel is used in all kinds of synthesis operations, and has an important value. Also, the grey algebra operation properties of ${\hat{⊗}}_{g^{o}}$ are discussed, and the relevant algorithm is proved. According to the gradation function of grey language and the definition of the kernel and grey scale, the grey language scoring function based on the kernel and grey scale is studied in this paper.

**Definition** **15.** *Assume* glh∈GLH, $GLH=\left\{\left(H_{1},\mu_{1},v_{1}),(H_{2},\mu_{2},v_{2}),\cdots (H_{l},\mu_{l},v_{l}\right)\right\}$

(5) $$S({\hat{⊗}}_{g^{o}})=\left(\frac{1}{l}\sum_{k=1}^{l}{\hat{⊗}}_{k},\max(g_{k}{}^{0})\right)=\begin{array}{cc} \left(\frac{1}{l}\sum_{k=1}^{l}\mu_{i}\cdot IH_{k},\max(v_{k})\right) & k=1,2\cdots l \end{array}$$

$S({\hat{⊗}}_{g^{o}})$ is the score function of the grey language based on the kernel and grey scale.

## 4. Kernel and Grey Scale Group Decision Making Method Based on Grey Language Hesitant Fuzzy Information

### 4.1. Model Construction

Assume that there are m feasible schemes $A_{1},A_{2},\cdots ,A_{m}$ and n evaluation criteria of a hesitant fuzzy group decision making problem based on grey language. $w={\left(w_{1},w_{2},\cdots ,w_{n}\right)}^{T}$ is weight vector corresponding to the evaluation criteria, where $w_{i}$ represents the weight of $P_{i}$. Assume the ordered language evaluation set $H=(H_{0},H_{1},\cdots ,H_{{}^{T}})$. According to the evaluation set, l experts evaluate various scheme indexes. In the grey language hesitant fuzzy set, ⊗ belongs to the discrete grey numbers.

$$GLH=\left[\begin{array}{cccc} glh_{11} & glh_{12} & \ldots & glh_{1n}\\ glh_{21} & glh_{22} & \ldots & glh_{2n}\\ \ldots & \ldots & \ldots & \ldots \\ glh_{m1} & glh_{m2} & \ldots & glh_{mn} \end{array}\right]$$

### 4.2. Decision-Making Steps

Step 1. In this step, the decision matrix $P_{m\times n}={\left(p_{ij}\right)}_{m\times n}$ is normalized as $R_{m\times n}={\left(r_{ij}\right)}_{m\times n}$.

It is necessary to eliminate the influence of different physical quantities before making a decision and to normalize different types of index values. Generally speaking, the cost index value should be converted to the benefit index. In special cases, the benefit index value also needs to be converted to the cost index value.

Cost index value $\left(H_{i},\mu ,v\right)$ is converted to benefit index value.

(6) $$r_{ij}=(H_{T-i},\mu ,v)$$

Benefit index value $\left(H_{i},\mu ,v\right)$ is converted to cost index value:

(7) $$r_{ij}=(H_{T-i},\mu ,v)$$

Step 2. Data supplement. Torra studied the hesitancy faced by experts in group decision making and proposed a hesitant fuzzy set suitable for group decision making.

Add the maximum membership scale to the hesitant fuzzy element when the decision maker has risk preference.

Add the minimum membership scale to the hesitant fuzzy element when the decision maker is risk-averse and pessimistic.

Step 3. Transform grey language data into a score function of kernel and grey scale through the Formula (4).

Step 4. Calculate grey correlation degree and grey scale.

**Definition** **16.** $\hat{⊗}$ *is the kernel part of score function of grey language element based on kernel and grey scale. The grey correlation coefficient* $\zeta_{i}(j)$ *and grey correlation degree* γ(j) *between the empirical judgment value and the reference weight value of each evaluation index are obtained by the following formula*:

*Make* $D=\left|{\hat{⊗}}_{i}(j)-{\hat{⊗}}_{0}(j)\right|$, *and*

(8) $$\xi_{i}^{}(j)=\frac{\underset{i}{\min}\;\underset{j}{\min}\;D+\rho \;\underset{i}{\max}\;\underset{j}{\max}\;D}{D+\rho \;\underset{i}{\max}\;\underset{j}{\max}\;D}$$

(9) $$\gamma (i)=\frac{1}{n}\sum_{j=1}^{n}\xi_{i}^{}(j),\begin{array}{cc} & 1\leq i\leq m \end{array}$$

*where*, ρ *is the resolution ratio*, ρ∈(0,1), *generally*, ρ=0.5. *The correlation degree of each sequence directly reflects the degree to which each evaluation is close to the optimal ideal scheme*.

**Definition** **17.** *Assume* $g^{0}(j_{\max})$ *as the maximum grey scale of kernel value in* j *column and* $g^{0}(i_{\max})$ *as the maximum grey scale of kernel value in* i *row*.

(10) $$g^{0}(i)=\max(g^{0}(j_{\max}),g^{0}(i_{\max}))\begin{array}{cc} & 1\leq j\leq n \end{array}$$

(11) $$\gamma_{g^{o}}(i)={\left(\gamma_{\left(g1^{o}\right)}(1),\gamma_{\left(g2^{o}\right)}(2),\cdots ,\gamma_{{}_{\left(gn^{o}\right)}}(m)\right)}^{T}\;\mathrm{Where},\;1\leq i\leq m$$

Step 5. The schemes are sorted according to the grey correlation degree and grey scale. The following principles should be followed:

(1) When γ(i)>γ(m), i≻m;

(2) When γ(i)=γ(m), and $g^{0}(i)>g^{0}(m)$, i≺m;

(3) When γ(i)=γ(m), and $g^{0}(i)=g^{0}(m)$, i≈m.

## 5. Example Analysis

As an economic development mode harmonious with the environment, the circular economy has become an important way to promote the comprehensive development ability of an industrial chain. Many industrial chains have carried out ecological transformations under the guidance of circular economy idea. The sustainable development of an industrial chain based on the circular economy is a complicated evaluation process, with uncertainty and multiple objectives. Evaluation of the sustainable development ability of an industrial chain based on the circular economy aims to study the optimization function of the circular economy on the design and reconstruction of the industrial chain, and to analyze the comprehensive development level and potential of the industrial chain from the perspective of the circular economy. The evaluation system of the sustainable development ability of an industrial chain based on the characteristics of the circular economy can provide scientific and effective means for the operation, management and improvement of the industrial chain. Therefore, it is necessary to establish an industrial chain sustainable development capacity evaluation index system covering four aspects of economic development: ($P_{1}$), resource utilization ($P_{2}$), environmental protection ($P_{3}$) and green management ($P_{4}$), so as to scientifically and objectively reflect the sustainable development capacity of an industrial chain [46]. Four industries $A_{1},A_{2},A_{3},A_{4}$ are assumed to be compared in the sustainable development of the circular economy industry chain in a certain area. Three experts evaluate and select $H=\left\{\begin{array}{l} H_{0}=\mathrm{very}\;\mathrm{poor},H_{1}=\;\mathrm{poor},H_{2}=\mathrm{relatively}\;\mathrm{poor},H_{3}=\mathrm{general}\\ H_{4}=\mathrm{good},H_{5}=\mathrm{relatively}\;\mathrm{good},H_{6}=\mathrm{very}\;\mathrm{good} \end{array}\right\}$ as the language evaluation term set. The evaluation value is represented by grey language value, forming the grey language hesitant fuzzy decision matrix in Table 1.

### 5.1. Method 1

Step 1. Normalize decision making matrix $P_{m\times n}={\left(p_{ij}\right)}_{m\times n}$ as $R_{m\times n}={\left(r_{ij}\right)}_{m\times n}$. All indexes in this example are benefit indexes, so they are omitted.

Step 2. Supplement data. Since there are three experts in this example, none of whom has abstained or given the same score, it is not necessary to supplement the data, and this step is omitted.

Step 3. Transform grey language data into the score function of the kernel and grey scale through Formula (5), and Table 2 is obtained.

Step 4. Calculate grey correlation degree and grey scale.

$$\xi_{i}^{}(j)=\left[\begin{array}{l} 0.5942\;0.5062\;0.5256\;0.3333\\ 0.7069\;0.6212\;0.5325\;0.4824\\ 1.0000\;1.0000\;1.0000\;1.0000\\ 0.9318\;0.5616\;0.6212\;0.6613 \end{array}\right]$$

$$\gamma (i)={\left(0.4898,0.5857,1.0000,0.6940\right)}^{T}$$.

$$\gamma_{g^{o}}(i)={\left(0.4898_{\left(0.50\right)},0.5857_{\left(0.40\right)},1.0000_{\left(0.30\right)},0.6940_{\left(0.40\right)}\right)}^{T}$$.

Step 5. Rank the schemes according to the grey correlation and grey scale of each industry. Obviously, $A_{3}≻A_{4}≻A_{2}≻A_{1}$.

### 5.2. Comparison Method 2

The following steps can be obtained according to the traditional decision making method in grey language.

Step 1. Normalize decision making matrix $P_{m\times n}={\left(p_{ij}\right)}_{m\times n}$ as $R_{m\times n}={\left(r_{ij}\right)}_{m\times n}$. All indexes in this example are benefit indexes, so they are omitted.

Step 2. Aggregate data $r_{ij}$ through Formula (1) to obtain $z_{ij}$, as shown in Table 3.

Step 3. Calculate the score function $S(a_{ij})$ of $z_{ij}$ through Formula (2), as shown in Table 4.

Step 4. Calculate grey correlation coefficient based on $S(a_{i})$ to obtain index weight w(j).

$$\xi_{i}^{}(j)=\left[\begin{array}{l} 0.4339\;0.3667\;0.3333\;0.3730\\ 0.5274\;0.4159\;0.3628\;0.5911\\ 1.0000\;1.0000\;1.0000\;0.3952\\ 0.5659\;0.3914\;0.5027\;1.0000 \end{array}\right]$$

$$w={\left(0.2729,\;0.2348,\;0.2375\;,0.2548\;\right)}^{T}$$

Step 5. Calculate the comprehensive criterion values of each scheme through the GLHFWAA operator.

$$\begin{array}{l} Z_{1}=0.2729\times (H_{3.6667},0.6364,0.4)+0.2348\times (H_{3},0.8,0.4)+0.2375\times (H_{3},0.6889,0.5)\\ +0.2548\times (H_{2},0.7,0.4)=(H_{2.9272},0.6996,0.5) \end{array}$$

Similarly,

$$Z_{2}=(H_{3.4174},0.8008,0.4),Z_{3}=(H_{4.1446},0.7537,0.3),Z_{4}=(H_{3.8417},0.7681,0.4).$$

Step 6. Compare $Z_{i}(i=1,2,3,4)$ and sort the schemes.

$$\begin{array}{l} IH_{2.9272}\times 0.6697\times (1-0.5)<IH_{3.4174}\times 0.8008\times (1-0.4)<IH_{3.8417}\times 0.7681\times (1-0.4)\\ <IH_{4.1446}\times 0.7535\times (1-0.3) \end{array}$$

$Z_{1}<Z_{2}<Z_{4}<Z_{3}$. Therefore, industry $A_{3}$ is the optimal, followed by industry $A_{4}$ and industry $A_{1}$ is the worst. 

### 5.3. Comparison Method 3

Step 5 can also be used to continue the decision making after decision step 4 in method 2.

Step 5. Select the grey language $S(a_{ij})$ decision matrix and grey correlation weight w(j) by using the multiple linear weighting method. The comprehensive criterion value of each scheme is calculated through the following formula:

$$Z_{i}=\sum_{j=1}^{n}S(a_{ij})\times w(j)$$

$$\begin{array}{l} =\left[\begin{array}{cccc} \begin{array}{l} 1.4000\;\\ 1.7800\;\\ 2.6133\;\\ 1.9000\; \end{array} & \begin{array}{l} 1.4400\\ 1.7400\;\\ 3.0462\;\\ 1.6000\; \end{array} & \begin{array}{l} 1.0333\;\\ 1.2600\;\\ 2.8933\;\\ 1.9733\; \end{array} & \begin{array}{l} 0.8400\;\\ 1.7600\;\\ 0.9800\;\\ 2.4033\; \end{array} \end{array}\right]\times {\left(0.2729,\;0.2348,\;0.2375\;,0.2548\;\right)}^{T}\\ ={\left[1.1796\;1.6420\;2.3653\;1.9752\;\right]}^{T} \end{array}$$

$Z_{1}<Z_{2}<Z_{4}<Z_{3}$, which is consistent with the sorting obtained through methods 1 and 2. By comparison, the grey scoring function used in method 1 is more reasonable and simple. Method 3 is simpler than method 2. On the whole, it can be concluded that method 1≻ method 3 ≻ method 2.

## 6. Conclusions

This paper studied the grey language decision making method, and defined the scoring function of grey language based on the kernel and grey scale which is more suitable for uncertain decision making. Moreover, a group decision-making method based on the grey correlation degree of the kernel and grey scale was proposed. 

In comparison, the method is more reasonable than traditional methods by introducing the kernel and grey scale to avoid adding subjective data, thereby reducing information distortion; in addition, the grey relational degree decision method does not need to calculate the index weight, and hence to some extent this method is more convenient. Of course, it is hard to avoid the lack of depth only through a comparison of three different decision methods of the same example, so we will conduct systematic comparative research on data simulation in future research so as to improve the credibility of the comparison.

## Figures and Tables

**Table 1:** The values of the grey language hesitant fuzzy decision matrix.

**Industries**	**Attribute** ($P_{1}$)	**Attribute** ($P_{2}$)
$A_{1}$	$\left\{\left(H_{3},0.6,0.3),(H_{4},0.6,0.4),(H_{4},0.7,0.4\right)\right\}$	$\left\{(H_{3},0.7,0.4),(H_{3},0.8,0.4),(H_{3},0.9,0.3),\right\}$
$A_{2}$	$\left\{\left(H_{3},0.7,0.4),(H_{3},0.8,0.4),(H_{4},0.9,0.1\right)\right\}$	$\left\{\left(H_{3},0.9,0.1),(H_{4},0.7,0.4),(H_{4},0.8,0.2\right)\right\}$
$A_{3}$	$\left\{\left(H_{4},0.7,0.2),(H_{5},0.6,0.2),(H_{5},0.8,0.1\right)\right\}$	$\left\{\left(H_{4},0.7,0.2),(H_{5},0.6,0.2),(H_{6},0.9,0.2\right)\right\}$
$A_{4}$	$\left\{\left(H_{3},0.6,0.2),(H_{4},0.8,0.4),(H_{5},0.9,0.3\right)\right\}$	$\left\{\left(H_{3},0.7,0.4),(H_{4},0.6,0.4),(H_{5},0.7,0.4\right)\right\}$
**Industries**	**Attribute** ($P_{3}$)	**Attribute** ($P_{4}$)
$A_{1}$	$\left\{\left(H_{2},0.8,0.5),(H_{3},0.6,0.4),(H_{4},0.7,0.4\right)\right\}$	$\left\{\left(H_{2},0.8,0.3),(H_{2},0.6,0.4),(H_{2},0.7,0.4\right)\right\}$
$A_{2}$	$\left\{\left(H_{3},0.6,0.2),(H_{3},0.7,0.4),(H_{3},0.8,0.3\right)\right\}$	$\left\{\left(H_{3},0.7,0.3),(H_{3},0.9,0.4),(H_{4},0.8,0.3\right)\right\}$
$A_{3}$	$\left\{\left(H_{4},0.8,0.3),(H_{4},0.8,0.2),(H_{5},0.7,0.2\right)\right\}$	$\left\{\left(H_{4},0.9,0.3),(H_{5},0.8,0.2),(H_{6},0.8,0.1\right)\right\}$
$A_{4}$	$\left\{(H_{3},0.8,0.1),(H_{3},0.6,0.2),(H_{4},0.8,0.1),\right\}$	$\left\{\left(H_{3},0.9,0.2),(H_{4},0.9,0.3),(H_{5},0.8,0.2\right)\right\}$

**Table 2:** ${\hat{⊗}}_{g^{o}}$ form of grey hesitant fuzzy decision matrix.

**Industries**	**Attribute** ($P_{1}$)	**Attribute** ($P_{2}$)
$A_{1}$	2.3333 _(0.40)_	2.4000 _(0.40)_
$A_{2}$	2.7000 _(0.40)_	2.9000 _(0.40)_
$A_{3}$	3.2667 _(0.20)_	3.7333_(0.20)_
$A_{4}$	3.1667 _(0.40)_	2.6667_(0.40)_
**Industries**	**Attribute** ($P_{3}$)	**Attribute** ($P_{4}$)
$A_{1}$	2.0667 _(0.50)_	1.4000 _(0.40)_
$A_{2}$	2.1000 _(0.30)_	2.6667 _(0.40)_
$A_{3}$	3.3000 _(0.30)_	4.1333 _(0.30)_
$A_{4}$	2.4667 _(0.20)_	3.4333 _(0.30)_

**Table 3:** $z_{ij}$ of grey language decision matrix.

**Industries**	**Attribute** ($P_{1}$)	**Attribute** ($P_{2}$)
$A_{1}$	$\left\{\left(H_{3.6667},0.6364,0.4\right)\right\}$	$\left\{\left(H_{3},0.8,0.4\right)\right\}$
$A_{2}$	$\left\{\left(H_{3.3333},0.89,0.4\right)\right\}$	$\left\{\left(H_{3.6666},0.7909,0.4\right)\right\}$
$A_{3}$	$\left\{\left(H_{4.6667},0.7,0.2\right)\right\}$	$\left\{\left(H_{5},0.7615,0.2\right)\right\}$
$A_{4}$	$\left\{\left(H_{4},0.7917,0.4\right)\right\}$	$\left\{\left(H_{4},0.6667,0.4\right)\right\}$
**Industries**	**Attribute** ($P_{3}$)	**Attribute** ($P_{4}$)
$A_{1}$	$\left\{\left(H_{3},0.6888,0.5\right)\right\}$	$\left\{\left(H_{2},0.7,0.4\right)\right\}$
$A_{2}$	$\left\{\left(H_{3},0.7,0.4\right)\right\}$	$\left\{\left(H_{3.6667},0.8,0.4\right)\right\}$
$A_{3}$	$\left\{\left(H_{5},0.8267,0.3\right)\right\}$	$\left\{\left(H_{2},0.7,0.3\right)\right\}$
$A_{4}$	$\left\{\left(H_{3.3333},0.74,0.2\right)\right\}$	$\left\{\left(H_{4},0.8583,0.3\right)\right\}$

**Table 4:** $S(a_{ij})$ decision matrix of grey language hesitation fuzzy.

Industries	Attribute ($P_{1}$)	Attribute ($P_{2}$)	Attribute ($P_{3}$)	Attribute ($P_{4}$)
$A_{1}$	1.4000	1.4400	1.0333	0.8400
$A_{2}$	1.7800	1.7400	1.2600	1.7600
$A_{3}$	2.6133	3.0462	2.8933	0.9800
$A_{4}$	1.9000	1.6000	1.9733	2.4033
